# Plasma variations in stress markers: Clinical trial of two anesthetics used in regional block in the extraction of impacted inferior third molars

**DOI:** 10.4317/medoral.19362

**Published:** 2013-12-07

**Authors:** Iciar Arteagoitia, Mercedes Zumarraga, Ricardo Dávila, Luis Barbier, Gorka Santamaría, Joseba Santamaria

**Affiliations:** 1 Stomatology I Department, University of the Basque Country, UPV/EHU, Bizkaia, Spain, Grupo de Alto Rendimiento Académico UPV/EHU IT821-13; 2 Research Department of Neurochemistry, Bizkaia Mental Health Network, Basque Health Service-Osakidetza, Zamudio Hospital, Bizkaia, Spain; 3 Maxillofacial Surgery Department, BioCruces Health Research Institute, Cruces University Hospital, Bizkaia, Spain. Stomatology I Department, UPV/EHU, Grupo de Alto Rendimiento Académico IT821-13; 4 Private Practice. Postgraduate Professor, University of the Basque Country, UPV/EHU

## Abstract

Objectives: Was to evaluate the effect of different regional anesthetics (articaine with epinephrine versus prilocaine with felypressin) on stress in the extraction of impacted lower third molars in healthy subjects. Sutdy Desing: A prospective single-blind, split-mouth cross-over randomized study was designed, with a control group. The experimental group consisted of 24 otherwise healthy male volunteers, with two impacted lower third molars which were surgically extracted after inferior alveolar nerve block (regional anesthesia), with a fortnight’s interval: the right using 4% articaine with 1:100.000 epinephrine, and the left 3% prilocaine with 1:1.850.000 felypressin. Patients were randomized for the first surgical procedure. To analyze the variation in four stress markers, homovanillic acid, 3-methoxy-4-hydroxyphenylglycol, prolactin and cortisol, 10-mL blood samples were obtained at t = 0, 5, 60, and 120 minutes. The control group consisted of 12 healthy volunteers, who did not undergo either extractions or anesthetic procedures but from whom blood samples were collected and analyzed in the same way. Results: Plasma cortisol increased in the experimental group (multiple range test, P<0.05), the levels being significantly higher in the group receiving 3% prilocaine with 1:1.850,000 felypressin (signed rank test, p<0.0007). There was a significant reduction in homovanillic acid over time in both groups (multiple range test, P<0.05). No significant differences were observed in homovanillic acid, 3-methoxy-4-hydroxyphenylglycol or prolactin concentrations between the experimental and control groups. Conclusions: The effect of regional anesthesia on stress is lower when 4% articaine with 1:100,000 epinephrine is used in this surgical procedure.

** Key words:**Stress markets, epinephrine versus felypressin.

## Introduction

Local and regional anesthetics are believed to be the drugs most frequently used in dentistry. It has been estimated that about 1,500 cartridges of anesthetic are used per dentist per year. Extrapolating this to dentists worldwide suggests that anesthetic block in dental clinics is probably the most widely used anesthetic technique for oral surgical procedures. For this, 4% articaine with 1:100,000 epinephrine and 3% prilocaine with 1:1.850.000 felypressin are among the most frequently used agents. Monitoring by drug control agencies indicates that these drugs can be used safely and effectively (1). Little is known, however, about their effect on levels of patient stress.

We selected extraction of impacted inferior third molars as the surgical technique in which to assess the variations in plasma levels of four stress markers: homovanillic acid (p-HVA); 3-methoxy-4-hydroxyphenylglycol (p-MHPG); prolactin (p-prolactin) and cortisol (p-cortisol). In controlled experimental conditions, p-HVA is the most reliable indicator of central dopaminergic activity in plasma (2-5); while p-MHPG is the main plasmatic norepinephrine metabolite and reflects peripheral noradrenergic activity (4). Levels of p-prolactin vary as a consequence of physical and psychological stress; furthermore, it is an indirect measurement of dopaminergic activity (3-5). Lastly, p-cortisol indicates hypothalamic-pituitary-adrenal-axe activity, which undergoes alterations in situations of stress (4,6).

The objective of this study was to clinically assess the effect of regional anesthetic block on stress levels. For this, we used two different vasoconstrictors in combination with an anesthetic (4% articaine with 1:100,000 epinephrine and 3% prilocaine with 1:1.850,000 felypressin) on levels of p-HVA, p-MHPG, prolactin, and cortisol in the extraction of impacted inferior third molars in otherwise healthy subjects.

## Material and Methods

We designed a prospective single-blind, split-mouth crossover randomized study, with a control group. The study was performed in accordance with the Declaration of Helsinki and the International Conference on Good Clinical Practice. The study protocol and informed consent forms were approved by Cruces Ethics Committee. All subjects were fully informed of the nature of the research, and gave their written consent to participate in the study. A registration number for this study is not available as it was designed before the enactment of the International Committee of Medical Journal Editors Guidelines.

We tested the null hypothesis that lower third molar exodontias cause no variations on serum markers of stress. If the null hypothesis is rejected we test whether both anesthetics have the same effect on serum stress markers.

The eligible population was all patients referred to the Department of Oral and Maxillofacial Surgery at Cruces University Hospital for the extraction of two impacted lower third molars with inferior alveolar nerve block (regional anesthesia). Inclusion criteria were: being a man between 18 and 25 years old, who was a non-smoker and had taken no medication in the previous month, with two lower third molars completely covered by mucosa and partially by bone.

The experimental group (EG) consisted of 24 otherwise healthy male volunteers, in the required age range (x=20.67 years; SD=1.84), with two lower third molars totally covered by mucosa and partially by bone that were subsequently surgically extracted under inferior alveolar nerve block anesthesia. These 24 patients were recruited and underwent surgery consecutively. They had their two lower third molars extracted a fortnight apart, the right lower third molar being extracted using 4% articaine with 1:100,000 epinephrine, and the left lower third molar using 3% prilocaine with 1:1.850,000 felypressin. The patients were randomized for the first surgical procedure. The control group (CG) consisted of 12 healthy male volunteers in the same age range (x= 21.03 years; SD=1.95). This group did not undergo either lower third molar extractions or anesthetic procedures; however, blood samples were collected on two occasions a fortnight apart (hereon, day 1 and day 2).

The setting and procedure for obtaining the blood samples were the same for both the experimental and control groups. The procedure commenced at the same time, 8.30 a.m., the participants having fasted since 12 p.m. the previous night. Venipuncture was performed, inserting a permanent catheter in the radial vein of the left arm. Once inserted, a 10-mL blood sample (t0´) was collected in a Vacutainer® tube.

The patients in the EG were immediately anesthetized to block the lower dental nerve plus the buccal nerve with 3.6 mL of either articaine with epinephrine or prilocaine with felypressin. A second 10-mL blood sample was collected 5 minutes after injecting the anesthetic (t5´). Surgery was carried out immediately thereafter, always by the same maxillofacial surgeon using the standard procedure: surrounding flap, ostectomy, odontosection, extraction and suture (7). The surgical time was measured from the mo-ment of the first incision until the last suture was cut (x=7.56 min; SD=1.98). In all cases, the surgery took less than 15 minutes, no patients complained of pain, and there were no cases of neurologic injury. Once surgery had been completed, the patient remained in the chair in a supine position, whilst two further 10-mL blood samples were extracted at 60 (t60´) and 120 (t120´) min-utes from commencing infiltration of the anesthetic.

Though participants in the CG underwent neither anesthesia nor lower third molar extractions, 10-mL blood samples were col-lected at t0´, t5´, t60´ and t120´, under the same environmental conditions, and this was done on two days a fortnight apart (day 1 and day 2).

Plasma was extracted from the blood samples by centrifugation (Beckman model TJ-6 centrifuge). Subsequently, 50 mL of sodium metabisulphite (50 mg/mL) was added to the plasma and the mixture was separated into five vials per sample (0.75- mL MICRO vials Freezly, SIGMA). The samples were stored in liquid nitrogen, until analysis.

The p-MHPG and p-HVA levels were measured using high-pressure liquid chromatography coupled with coulometric detection (8), while p-cortisol and p-prolactin levels were determined by a radioimmunoassay procedure (Diagnostic Product Corporation).

Statistical analysis was performed using Statgraphics Centurion XVI. The sample size was not based on formal power calculations. The mean variations in each of the groups in plasma levels of the markers at the four time points (t0´, t5´, t60´ and t120´) were studied with analysis of variance, the Kruskall-Wallis test and the multiple range test (MRT). Further, variations between the groups in the marker plasma levels at the same time points were studied using the Student’s t-test, analysis of variance, the multiple range test and signed range test (SRT). Test for related samples were used as data were not independent. The level of significance was preset at 0.05.

## Results

The mean and standard deviation of the plasma concentration of the four stress markers are summarized in  Table 1 to  Table 4.

**Table 1:**
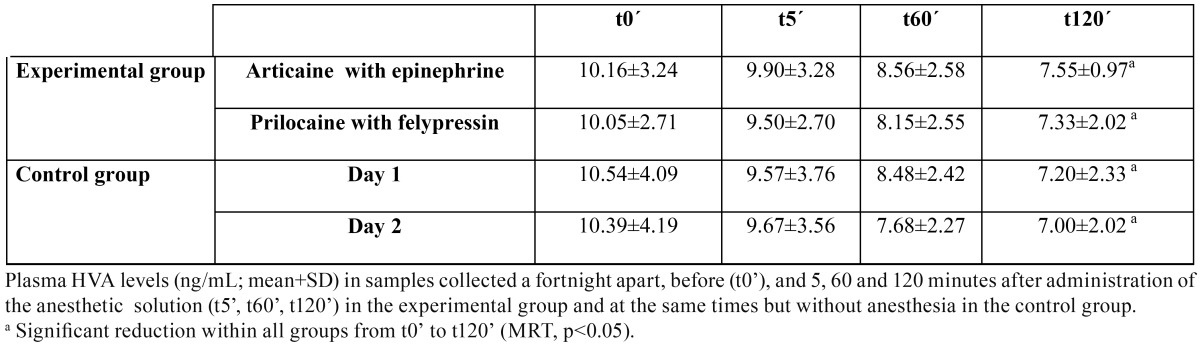
Mean plasma levels of homovanillic acid.

**Table 2:**
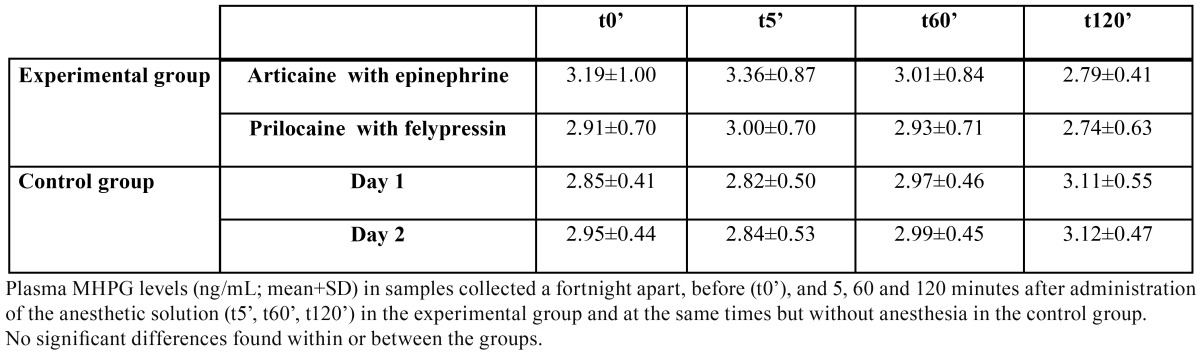
Mean plasma levels of 3-methoxy-4-hydroxyphenylglycol.

**Table 3:**
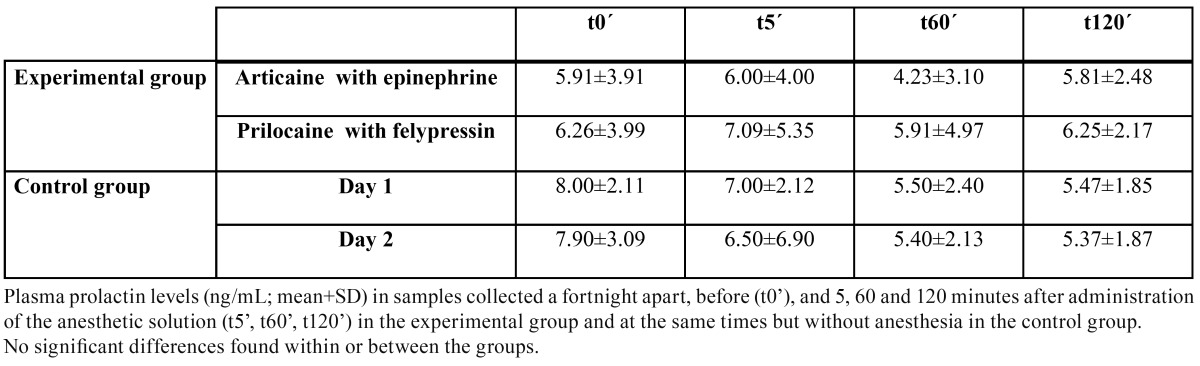
Mean plasma levels of prolactin.

**Table 4:**
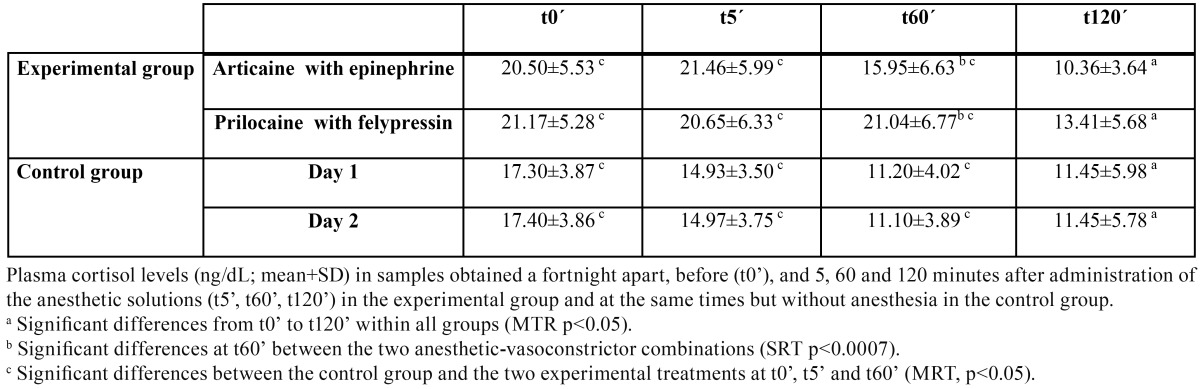
Mean plasma levels of cortisol.

A significant reduction in p-HVA levels was observed in all the subgroups as time passed ( Table 1; MRT, *p*<0.05). There were, however, no significant differences in the mean p-HVA levels at any of the time points between the experimental and control groups, or between the two experimental subgroups in which different anesthetic/vasoconstrictor combinations were used.

Regarding p-MHPG levels ( Table 2), there were no significant differences within or between the groups at any time, rather the levels of this marker seemed to remain fairly stable in each individual throughout. Further, the p-MHPG levels did not correlate with those of the p-HVA.

Similarly, there were no significant differences in p-prolactin levels at any time, either within or between the groups ( Table 3). It must, however, be borne in mind the participants were healthy males, with very low values, close to the limits of detection of the technique used.

Lastly, we observed a trend for p-cortisol levels to fall in all the groups over time ( Table 4). Comparing the groups, there were significant differences between the experimental and control groups at t0´, t5´ and t60´ (MRT, *p*<0.05), though not at t120´. Further, comparing p-cortisol levels between the two subgroups in which different anesthetic/vasoconstrictor combinations were used, the mean values were significantly higher at t60´ in the group receiving prilocaine as anesthesia (signed rank test, *p*<0.001). The same trend persisted at t120´, although at this point the difference was not significant.

## Discussion

The variations in levels of p-HVA, p-MHPG and p-prolactin observed in this study did not differ significantly between the control and experimental groups. On the other hand, the variations in plasma cortisol were significantly different, the level being higher in the experimental than the control group at all the time points studied (t0, t5, t60 and t120´). Further, the anesthetic/vasoconstrictor combination used for the regional block influenced cortisol: levels were higher in patients given 3% prilocaine with 1:1.850,000 felypressin, which corresponds clinically to higher levels of stress when this combination is used.

Millions of dental interventions are carried out every day across the world using vasoconstrictors in combination with anesthetics. Though it is known that these interventions cause stress, little is known about how this stress is affected by different anesthetic approaches. Hence, we set out to explore whether anesthetics with vasopressors affected patient stress levels and, if so, whether the effect was the same with all anesthetics. For this, we considered the extraction of impacted inferior third molars under regional anesthesia, namely inferior alveolar nerve block. This surgical procedure is reproducible and has been used previously as a research model to assess the efficacy of drugs (9), hemodynamic changes (10) and stress markers (11).

The main purpose of the split-mouth design is to control for all confounding factors related to differences between subjects by making within-patient comparisons, rather than between-patient comparisons; in this way, the experimental error variance can be reduced, and this means a statistically more powerful study. Unfortunately, comparisons made on a within-patient basis also have potential disadvantages. For this reason, we included a control group in which no treatment was carried out to analyze variation in the markers.

Human response to surgical stress is characterized by a massive release of neuroendocrine hormones provoking many metabolic and physiological changes (4). To explore a range of these changes, we studied four markers that have previously been associated with various situations of stress: p-cortisol, p-prolactin, p-HVA and p-MHPG (2-6).

The circadian rhythm of p-cortisol and p-prolactin are well known. Further, it has been demonstrated (12) that p-HVA levels tend to fall over the course of the morning. Our methodology allowed for these patterns since the study, in both the experimental and control groups, started at 8.30 a.m. Furthermore, our model commenced in a maximum stress situation, which converts to one of rest, once surgery is over. This is a key characteristic of the study, other authors studying the stress response having initiated the experiment in a rest situation and subsequently provoked stress over the course of the morning (3).

In our research, we decided to study these stress markers in plasma, as we believe that the measurements are more accurate than in urine, saliva or tears (11). We recognize, however, that venipuncture in itself could be a source of stress. If this were to be the case, our design minimizes the possible associated bias, as experimental and control groups are subjected to the same blood collection procedure. On the other hand, to minimize the stress potentially caused we decided to use the Vacutainer® technique, as this meant that only one puncture was required to collect the four blood samples. We are unable to confirm the hypothesis of Grayson *et al.* (13) that an increase in p-prolactin and p-HVA levels is observed in association with the stress of venipuncture, as independent of any intervention the levels of these markers in blood peak in the early hours of the morning and progressively decrease throughout the day, due to the circadian rhythm (5,12).

The absence of significant differences in the concentrations of p-HVA between the experimental and control groups indicates that the surgical procedure has no impact on this marker. The norepinephrine metabolite, p-MHPG, had a stable concentration throughout the time period in all the groups; nevertheless, its concentration was slightly higher at t5´ and t60´ in the group administered articaine with epinephrine. These results are consistent with data from other authors affirming that p-MHPG variations are not attributable to the circadian rhythm (14). No significant differences were observed within or between groups when using catecholamine or vasopressin analogues as a vasoconstrictor.

As was to be expected, p-cortisol levels did vary, showing a significant reduction in concentration over time, both in control and experimental groups. There were, however, significant differences between the groups, with higher concentrations in the experimental groups than the controls. Indeed, the data suggest that p-cortisol in the experimental groups was influenced by the stress associated with the surgical procedure. Further, the significant increase in p-cortisol at 60 minutes in those given prilocaine with felypressin ( Table 4) suggests that this group had clinically higher levels of stress throughout the study period than those given articaine with epinephrine. These results confirm previous findings (6).

In certain circumstances, the prolactin hormone under dopaminergic control may be a stress indicator. Further, p-prolactin levels have been reported to increase in stressful situations (5); nevertheless, our results have not enabled us to confirm these previous findings. In the experimental groups, plasma levels at t0´ were lower than those in the control group. There was a slight increase at t5´ in the experimental groups, this being more pronounced in the articaine group. Subsequently, levels fell in all the groups throughout the study period. This longer-term trend, which was more pronounced in the controls, may be due to the circadian rhythm and associated decrease in plasma levels throughout the morning. One of the limitations in the analysis of prolactin is due to its plasma concentrations being on the technical limits of detection, all participants being men. We chose to limit the study to men to avoid the confounding variable of sex. Furthermore, there is wide range of values, particularly in the experimental groups, as reflected in the large SD.

The types of local anesthetics used in our study, one group receiving epinephrine and the other felypressin, were not seen to produce any differences in the markers studied, except in the case of p-cortisol that was lower in the epinephrine group at t60´.

In conclusion, firstly, in a healthy young population, the stress generated by lower third molar exodontias causes variations in p-cortisol levels, the best plasma marker of stress, but not in the other markers studied, namely p-HVA, p-MHPG and p-prolactin. Secondly, the use of articaine with epinephrine is associated with a smaller increase in cortisol levels, corresponding clinically to a lower level of stress. The effect of different anesthetic-vasoconstrictor combinations on stress should be considered in deciding which anesthetic approach to use.
